# The impact of COVID-19 on critical cardiac care and what is to come postpandemic

**DOI:** 10.2217/fca-2020-0093

**Published:** 2020-07-10

**Authors:** Justin Hall

**Affiliations:** ^1^iRhythm Technologies Ltd, Seat House, 56 London Road, Bagshot, Surrey, GU19 5HL, UK

**Keywords:** arrhythmias, atrial fibrillation, devices

As humans, we are programed to remember negative events over positive. Take the history of global health, for example. School curriculums and textbooks emphasize the terror and devastation caused by smallpox, cholera, yellow fever and polio, only to overlook the medical advancements that were developed in response.

Today we are facing a similar situation, with COVID-19 transforming the way the National Health Service (NHS) operates entirely. While the natural priority during this time has been to ensure public safety and survival, it has also helped to establish whether or not healthcare professionals are set up for success, including cardiologists. When a global pandemic hits, do they have access to the tools and technologies they need to continue the expert diagnosis, treatment and management of various heart conditions? Or are they having to compromise on maintaining normal levels of service?

According to a study from the European Society of Cardiology [1], the number of heart attack patients seeking urgent hospital care dropped by more than 50% during the early months of the pandemic. Some sufferers stayed away due to fears of catching the virus, while others wanted to avoid becoming yet another patient for the NHS to care for. Yet, by not acting quickly and seeking immediate medical treatment, many individuals put their lives at risk. In all areas of cardiac care, the ability to act almost immediately is key. The sooner an accurate diagnosis is made, the quicker a suitable treatment plan can be decided upon, and the likelihood of a full recovery significantly increases.

Add to this, the number of scheduled cardiac appointments that were missed during the first weeks of the outbreak – which could have detected the early signs of an arrhythmia or other serious heart conditions – and it is likely that the NHS could face a spike in the number of patients suffering with severe cardiac problems further down the line.

However, in an effort to mitigate this challenge and ensure that effective cardiac diagnosis can continue throughout this period, GPs and cardiac practitioners have adapted; shifting to video consultations and virtual clinics in order to continue treating patients. Meanwhile, providers of cardiac diagnostic tools have transformed their entire models in an attempt to ease some of the pressure currently being placed on the NHS. Despite some initial challenges in getting set up, we are now starting to see the benefits of these new approaches to cardiology.

Patients still feel as though they have access to the care and advice that they need, while practitioners are able to continue to support patients without increasing strain on their already stretched resources. These are the models that we need to focus on. Not only to get us through the current crisis, but to better future proof cardiology departments and their experts. We are already on the path to reshaping outdated ways of working, we just need to make sure innovation continues. And here’s how….

## Modernizing primary care

Across the healthcare industry as a whole, staff are looking at new ways of managing patients in the wake of COVID-19. Organizations are being asked to ‘lock in beneficial changes brought about since the pandemic began, including the rapid scaling of new technology-enabled service delivery options.’ [2].

In direct support of meeting the challenges that lie ahead, many providers of cardiac care and relevant technologies have adapted their offerings to make them more convenient and safer to access. Despite many of these new fulfillment models being set up at short notice, and without the benefit of extensive planning, they have suddenly become essential. Professionals have seen the day-to-day routines that they have followed for years being replaced by more agile and efficient methods, practices and procedures.

For example, the need to stay at home and meet social distancing guidelines, has driven a surge in telemedicine, with many GPs turning to online consultations in order to continue to effectively diagnose and treat cardiac patients without having to physically see them. In fact, it has been estimated that only 7–8% [3] of all NHS consultations are being carried out face-to-face at the present time.

With the majority of preliminary cardiac appointments now being carried out either digitally – via video platforms or remotely – via telephone, practitioners are able to significantly reduce the amount of time and admin associated with typical patient visits. Medical records can be easily accessed, consultations can run swiftly and most importantly, doctors can still determine what form of care to recommend.

The benefits of fewer face-to-face visits are already being widely seen by those within the healthcare industry, with some physicians [4] and even the Health Secretary, Matt Hancock [5], predicting that remote appointments are here to stay once the COVID-19 pandemic passes. While accelerating innovation has been the topic of many discussions and debates across the NHS in recent years, it has been far from the reality. Despite presenting itself under negative circumstances, we have finally seen the necessary steps being taken to fast-track the health service – and cardiac diagnosis and care – into the 21st century.

## Personalizing cardiology

It is not just healthcare professionals who have had to adapt to the current climate and change the way that they interact with patients. Following the redeployment of health centers and the reallocation of resources, providers of cardiac technologies have also had to adapt and come up with innovative methods to ensure that their life-saving devices and solutions are as accessible as possible to both patients and practitioners.

It is recognized that patients with cardiovascular diseases are among those most at risk of contracting coronavirus. In light of this, iRhythm have launched a direct to patient shipping model for its Zio service. This allows many high-risk individuals to access the critical cardiac care they need, without having to visit hospitals or leave their homes.

Thanks to this model, patients are able to apply single-use cardiac monitors themselves at home and still get the same results that they would receive following a clinical application. This means that the crucial reports produced are still both accurate and easy to read but involve minimal interaction between patients and healthcare staff, keeping everybody safe. Ultimately, it is helping to ensure that patients suffering with potential arrhythmias – and therefore at greater risk of suffering a stroke – can continue to access vital cardiac monitoring services without coronavirus fears getting in the way.

Without a vaccine there can be no silver-bullet solution to curing COVID-19. However, in recent weeks, many providers of health technologies and solutions have been forced to shift their way of thinking and as a result innovative new patient models have been born. These models are successfully delivering care to those that need it, while helping to reduce the strain placed upon other healthcare resources during this time and perhaps even beyond.

## Committing to a digitally enabled future

Despite a renewed focus on digital advancements in the healthcare space in recent years, the NHS has traditionally – and quite understandably – been slow to fully embrace innovation. Time and time again, rigorous testing, budget restraints and, arguably, a fear of the unknown have gotten in the way and made bringing some of the most cutting-edge cardiac technologies to market almost impossible. Valuable innovation is taking a long time to break through and, when it does, uptake and adoption is slow, complex and laborious.

When COVID-19 struck, it forced the entire healthcare system to be creative in its response. Since then, the need to mitigate its spread and continue to care for patients both with and without the virus has driven a surge in innovation. Those on the front line of cardiac care – GPs, medical practitioners, nurses and other healthcare professionals – have worked together with those behind the scenes – health tech providers and medical device manufacturers. This united front has enabled us to fight the virus’s effects and gain the upper hand while maintaining the highest levels of diagnosis and treatment.

Once the crisis is over, however, it is critical that we do not take five steps back. Instead, we must continue to drive much needed innovation across the entire healthcare sector, modernizing our digital infrastructure in order to further develop a future proof NHS. The key to driving this innovation, lies not only with those creating it, but with those that are expected to use it every day. If digital plans are to succeed post-COVID-19, personal and professional buy-in from medical professionals will be essential. Part of getting this buy-in will be ensuring that the NHS workforce is empowered to use any new technologies that come into play.

The current surge in remote appointments and video conferencing has made many in the industry rethink traditional training practices. Health Education England’s deputy medical director, Professor Simon Gregory, reflected in a recent blogpost that the delivery and assessment of GP training will need to ‘reflect the new norm’ and that general practice needs to ‘train for the future not the past’ moving forward [6]. This is the case for all new technologies brought to market in the future – a certain level of upskilling and digital training needs to take place in order to ensure that they can be used effectively. The medical professionals leading the charge in cardiac care need to feel supported both now and in the future. It is only then that innovation will be embraced throughout the industry.

## A new dawn in cardiology

The UK’s health service has shown immense resilience in the face of COVID-19, adapting practices and going above and beyond in order to save lives and restore a sense of normality. Although the devastation caused by the virus cannot be denied, it has opened many doors and created a platform for innovation and change within an industry that had found itself almost stagnate for some time. This time of hardship has also been a time of great creativity; with cardiac health tech providers and medical professionals rising to the challenge and altering well established and accepted systems and practices.

Moving forward, we must seek to carry this drive for innovation into our post-COVID-19 landscape. The lessons we have learnt and the successes we have had during this difficult period will help us to reach a new, digitally empowered age in cardiac care. This turning point in history will be vital in mitigating future crises and making the UK’s healthcare system sustainable long term.
